# Association between Traffic Related Air Pollution and the Development of Asthma Phenotypes in Children: A Systematic Review

**DOI:** 10.1155/2018/4047386

**Published:** 2018-12-02

**Authors:** Nelson Lau, Alex Norman, Mary Jane Smith, Atanu Sarkar, Zhiwei Gao

**Affiliations:** ^1^Department of Clinical Epidemiology, Faculty of Medicine, Memorial University of Newfoundland, Newfoundland and Labrador, Canada; ^2^Discipline of Pediatrics, Faculty of Medicine, Memorial University of Newfoundland, Newfoundland and Labrador, Canada; ^3^Division of Community Health and Humanities, Faculty of Medicine, Memorial University of Newfoundland, Newfoundland and Labrador, Canada

## Abstract

**Introduction:**

Traffic related air pollution (TRAP) has long been associated with the onset of childhood asthma. The relationship between TRAP exposure and the development of childhood asthma phenotypes is less understood. To better understand this relationship, we performed a systematic review of the literature studying childhood TRAP exposure and the development of childhood asthma and wheezing phenotypes (transient, persistent, and late-onset asthma/wheezing phenotypes).

**Methods:**

A literature search was performed in PubMed, Embase, and Scopus databases for current literature, returning 1706 unique articles. After screening and selection, 7 articles were included in the final review. Due to the low number of articles, no meta-analysis was performed.

**Results:**

TRAP exposure appears to be associated with both transient and persistent asthma/wheezing phenotypes. However, there was little evidence to suggest a relationship between TRAP exposure and late-onset asthma/wheezing. The differing results may be in part due to the heterogeneity in study methods and asthma/wheezing phenotype definitions, in addition to other factors such as genetics.

**Conclusion:**

TRAP exposure may be associated with transient and persistent asthma/wheezing phenotypes in children. The low number of studies and differing results suggest that further studies are warranted.

## 1. Introduction

Childhood asthma is the most common chronic disease in children, with estimated prevalence of 14% in children worldwide [1, 2]. This high prevalence is also associated with significant economic burden. Asthma in school-aged children in the United States alone is estimated to cost nearly $6 billion annually in healthcare expenditures [3]. Given the high burden of disease as well as the complex and heterogeneous nature of childhood asthma, it is essential to investigate further beyond the incidence and outcomes associated with childhood asthma [4].

Among the first studies to investigate the differences between childhood asthma symptoms was the Tucson Children's Respiratory Study, which identified 4 separate wheezing phenotypes based on the longitudinal pattern of wheezing that was observed [5]. These phenotypic groups were based on the age of wheezing onset and the duration of wheezing and included the following groups: (1) no wheezing, (2) early transient wheezing (wheezing before age of 3 but not at age of 6 years), (3) persistent wheezing (wheezing both before age of 3 and at age of 6 years), and (4) late-onset wheezing (no wheezing before age of 3 but wheezing by age of 6 years) [5]. The existence of these phenotypes has been supported by further studies, using methods such as latent class analysis and group based trajectory modelling [4, 6–8]. Currently, childhood asthma consists of many different phenotypes, each associated with differing clinical and genetic markers, risk factors, outcomes, and responses to medication [9, 10]. Thus, understanding the different clinical phenotypes of childhood asthma and wheeze may lead to several benefits in diagnosis and treatment. These include knowledge of probable outcomes and prognosis, personalized treatments for patients, and understanding how environmental exposures can modify the risk of developing different childhood asthma or wheezing phenotypes [11].

Numerous studies have found traffic related air pollution (TRAP) to be associated with the onset of childhood asthma [12–16]. These results are further supported by a systematic review that showed strong associations between exposure to black carbon (BC), NO_2_, PM_2.5_ (atmospheric particulate matter less than 2.5 *μ*m in diameter), and PM_10_ (atmospheric particulate matter less than 10.0 *μ*m in diameter) with the onset of childhood asthma [17]. The association between TRAP and different childhood asthma phenotypes is less understood. Earlier reviews have focused primarily on the association between TRAP and the onset of childhood asthma rather than the development of the asthma phenotype [17, 18]. Nevertheless, the effect of TRAP exposure on the development of different childhood asthma phenotypes may be significantly different. One study found no association between NO_2_ exposure and either early transient wheeze or persistent wheeze phenotypes in children [14]. These findings conflict with another study that found an association between childhood NO_2_ exposure and persistent wheeze in children [19]. These associations may also change with the pollutant being studied: although one study found no association between childhood NO_2_ exposure and early transient wheezing, it found an association between childhood PM_2.5_ exposure and early transient wheezing [14].

The purpose of this systematic review is to synthesize the results of observational epidemiological studies studying the association between TRAP exposure and the development of childhood asthma/wheezing phenotypes, namely, transient asthma/wheezing, late-onset asthma/wheezing, and persistent asthma/wheezing in children aged 0-18 years.

## 2. Methods

### 2.1. Selection Criteria

This systematic review was conducted in accordance with the Preferred Reporting Items for Systematic Reviews and Meta Analyses (PRISMA) statement for reporting systematic reviews and meta-analyses [24]. The review included cross-sectional, case-control, and cohort studies which studied the association between TRAP exposure and the development of childhood asthma phenotypes, namely, early-transient asthma, late-onset asthma, and persistent asthma in children aged 0-18 years.

Studies were included if they

1. were epidemiological or observational studies such as cross-sectional, cohort, or case-control studies;
2. had some measure of TRAP (CO, PM_2.5_, PM_10_, and NO_2_) exposure [25] for children within the early life period between fetal stage and age of 12 (through either modelling or direct measurement);
3. examined the association between TRAP exposure and development of asthma or wheeze outcomes when the child is aged 0-18 years;
4. explicitly included at least one type of asthma/wheezing phenotype (late-onset asthma/wheeze, persistent asthma/wheeze, and transient asthma/wheeze) in their outcomes.

Studies were excluded if they

1. measured TRAP exposure only when children were aged > 12 years;
2. were reviews, commentaries, experimental studies, letters to the editor, and so forth;
3. were studies that only examine the association between TRAP exposure and asthma development without specifying the phenotype or look at exacerbation of asthma/wheeze, allergies, and so forth as the outcome;
4. measured exclusively pollution exposure to non-TRAP pollutants, such as O3 or SO2;
5. were non-English-language studies.

No studies were excluded on the basis of publication year.

### 2.2. Health Outcomes

The primary health outcomes assessed were childhood asthma/wheezing phenotypes. Articles with either wheezing phenotypes or asthma phenotypes as outcomes were included for analysis. Although wheezing is a nonspecific symptom that is not always associated with childhood asthma, wheezing phenotypes have long been used to characterize the corresponding childhood asthma phenotypes [18, 26, 27]. To account for the differing follow-up times between studies, asthma/wheezing phenotypes were divided into 3 groups with the following modified definitions based on the Tucson Children's Respiratory Study [5]:

1. Transient asthma/wheezing: onset of asthma or wheezing before or at age of 3 and no asthma or wheezing after age 3
2. Persistent asthma/wheezing: onset of asthma or wheezing before or at age of 3, with evidence of asthma or wheezing after age of 3
3. Late-onset asthma/wheezing: onset of asthma or wheezing after age of 3

### 2.3. Search Strategy

Searches were performed in the PubMed, Embase, and Scopus databases for relevant articles. Search strings containing terms for “asthma,” “vehicle emissions,” and “children” were used. An example search string for PubMed is given below:

(“Asthma”[Mesh] OR asthma OR wheeze) AND (“Motor Vehicles”[Mesh] OR “Vehicle Emissions”[Mesh] OR traffic OR car OR truck OR bus OR motorcycle OR automobile OR vehicle OR exhaust) AND (“Child”[Mesh] OR “Infant”[Mesh] OR childhood OR children OR infant OR baby OR paediatric OR pediatric OR paediatrics OR pediatrics)

The search was performed in May 2018 and included papers published until May 2018.

### 2.4. Quality Assessment

The Critical Appraisal Skills Programme (CASP) checklist for cohort studies was used to assess the quality of applicable studies [28]. CASP consists of 12 questions used to evaluate the quality of cohort studies. The CASP criteria were used to evaluate cohort studies for (1) selection bias in the cohorts used, (2) measurement, classification, or recall bias in exposures, (3) measurement or classification bias in outcomes, (4) adjustment for appropriate confounders, (5) length and completeness of follow-up, and (6) potential validity of results. Two reviewers (N.L. and A.N.) independently assessed each article using the CASP criteria.

### 2.5. Data Extraction

Relevant information was extracted independently by two reviewers (N.L. and A.N.). Information was extracted from supplementary materials when deemed necessary. Disagreements on what information to extract were resolved via consensus by both reviewers. Extracted information included authors, study location, year of publication, study design, study population, pollutant and exposure information, asthma and wheezing phenotype definitions, and outcome data.

## 3. Results

### 3.1. Search Results

**Figure 1:**
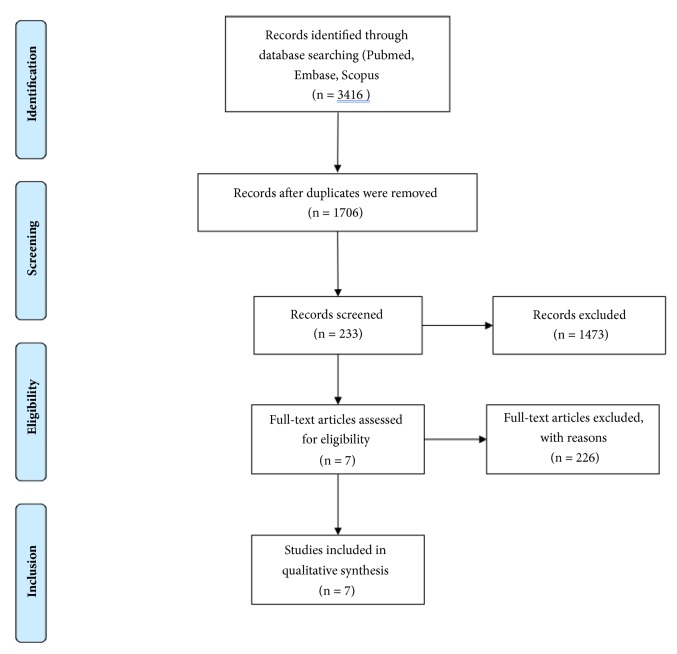
Preferred Reporting Item for Systematic Reviews and Meta-Analyses (PRISMA) flow diagram for article selection.

A literature search was conducted in the PubMed, Embase, and Scopus databases, yielding 1706 unique articles. After initial screening, 233 articles were chosen for full-text review, using the selection criteria and 7 articles were deemed suitable for inclusion [4, 14, 19–23].  Figure 1 represents the PRISMA flow diagram for article selection in this study.

### 3.2. Study Characteristics

The 7 studies included were published from 2007 to 2018, with all being cohort studies [4, 14, 19–23]. Among the 7 studies, one birth cohort was utilized twice in separate studies [14, 19]. 111 038 individuals across these 7 studies were included (duplicated cohorts were counted twice). The sample size in the included studies ranged from 2871 to 68 195 individuals. Studies were conducted in Canada, France, USA, Sweden, Netherlands, and Norway and were all English language studies. The length of follow-up varied among the 7 studies, with all studies starting from birth and the end of follow-up ranging from age 4-12. To estimate pollutant exposure, 3 studies utilized Land Use Regression (LUR) and 4 utilized dispersion modelling. CO, NO_2_, NO_x_, PM_2.5_, and PM_10_ were the traffic related air pollutants assessed. The number of studies measuring each individual pollutant is as follows:

- CO: 1 study
- NO_2_: 4 studies
- NO_x_: 3 studies
- PM_2.5_: 4 studies
- PM_10_: 1 study

Due to the differing follow-up times among the included studies, phenotypic definitions varied by study. 5 studies reported results in the form of odds ratios for asthma phenotype risk per unit of pollutant exposure (*μ*g/m^3^) [14, 19–21, 23]. One study, that by Sbihi et al., divided the cohort into quartiles based on exposure quartiles to the lowest quartile of pollutant exposure [4]. The last study, that by Pennington et al., reported asthma phenotype risk in the form of absolute risk difference between different exposure groups [22]. Complete study characteristics, including phenotypic definitions, can be found in Table 1, while the individual results for each study can be found in Table 2. Due to the low number of included studies, we were unable to conduct a meta-analysis.

### 3.3. Quality Assessment of Studies

All included studies were considered to be of sufficient quality for inclusion. The most common limitations identified from the CASP checklist were the potential for recall bias in studies where outcomes were reported via questionnaire and not adjusting for potential confounders.

### 3.4. Effect of CO on Childhood Asthma Phenotype Development

The sole study which measured CO exposure measured only persistent childhood asthma by age of 5 as a phenotypic outcome [22]. Prenatal exposure to CO was associated with an absolute risk increase of 3.5% for persistent asthma at age of 5. Exposure to CO during the 1st year of life was associated with an absolute risk increase of 3.9% for persistent asthma at age of 5.

### 3.5. Effect of NO_2_ on Childhood Asthma Phenotype Development

Of the four studies that measured the association between NO_2_ and the development of childhood asthma phenotypes, two reported associations for transient, persistent, and late-onset asthma/wheeze, one reported associations for transient and persistent wheeze, and one study reported solely the association for late-onset asthma [4, 14, 19, 21]. Two studies reported results from an identical study cohort (PIAMA) [14, 19].

Two of the studies that listed transient asthma/wheezing as an outcome found a significant association between NO_2_ exposure and transient wheezing [4, 19], with the third study reporting no significant association [14]. However, a significant association was reported by Sbihi et al. only when the second and fourth exposure quartiles were compared to the lowest quartile. No association was observed between NO_2_ exposure and transient wheezing when the third exposure quartile was compared to the lowest quartile [4].

Of the three studies that reported persistent asthma/wheezing, two studies reported no association between NO_2_ and persistent wheezing [14, 19]. The third study reported significant associations for the second and third exposure quartiles compared to the reference quartile; but the highest exposure quartile was not associated with persistent asthma [4].

Two of three studies found no association between NO_2_ and late-onset asthma/wheezing [14, 21]. Significant associations for the second and third exposure quartiles with late-onset asthma were reported by the third study, but the highest exposure quartile was not associated with late-onset asthma [4].

### 3.6. Effect of NO_x_ on Childhood Asthma Phenotype Development

Among the three studies that measured the association between NO_x_ exposure and the development of childhood asthma phenotypes, two studies studied transient wheezing, persistent wheezing, and late-onset wheezing as outcomes [20, 23]. One study studied solely persistent asthma [22].

Pennington et al. found that prenatal exposure to NO_x_ was associated with an absolute risk increase of 3.8% for persistent asthma at age of 5, while exposure to NO_x_ during the 1st year of life was associated with an absolute risk increase of 4.0% for persistent asthma [22].

Both studies which reported odds ratios for phenotypic outcomes found a significant association between NO_x_ exposure and persistent wheezing, with no association found with NO_x_ and either transient or late-onset wheezing [20, 23].

### 3.7. Effect of PM_2.5_ on Childhood Asthma Phenotype Development

Among the four studies which measured the association between PM_2.5_ exposure and the development of childhood asthma phenotypes, two studies studied transient wheezing, persistent wheezing, and late-onset asthma or wheezing as outcomes [4, 14]. One study reported associations for transient and persistent wheeze [19]. The final study contained solely persistent asthma as a phenotypic outcome [22]. Two studies, those by Brauer et al. and Gehring et al., reported results from an identical study cohort (PIAMA) [14, 19].

Pennington et al. found that prenatal exposure to PM_2.5_ was associated with an absolute risk increase of 4.4% for persistent asthma at age of 5 [22]. Exposure to PM_2.5_ during the 1st year of life was associated with an absolute risk increase of 4.5% for persistent asthma at age of 5.

Among the three studies which reported associations between PM_2.5_ and transient asthma or wheezing, two reported a significant association [14, 19]. The third one by Sbihi et al. reported a significant association between transient asthma and the second and third exposure quartiles [4]. However, the highest exposure quartile was not associated with transient asthma.

Two of three studies found no association between PM_2.5_ exposure and persistent wheezing or asthma phenotype [14, 19]. Sbihi et al. found that the second exposure quartile of PM_2.5_ was associated with persistent asthma, but there was no association between PM_2.5_ and the third and fourth quartiles [4].

Gehring et al. reported an association between PM_2.5_ and late-onset wheezing [14]. Sbihi et al. also reported that the second and third exposure quartiles were associated with late-onset asthma, although there was no association with the highest exposure quartile [4].

### 3.8. Effect of PM_10_ on Childhood Asthma Phenotype Development

Nordling et al. reported the association between PM_2.5_ exposure and the development of transient wheezing, persistent wheezing, and late-onset wheezing as outcomes [20]. No significant association was found between PM_10_ and any of the three wheezing phenotypes.

## 4. Discussion

Although previous studies have looked at TRAP and the onset of childhood asthma, to our knowledge this is the first attempt to systematically evaluate the available literature on the effect of TRAP exposure with the development of childhood asthma or wheezing phenotypes. 7 studies were included for final analysis. The results suggest that TRAP is associated with the development of childhood transient and persistent asthma/wheezing phenotypes but may not be associated with late-onset asthma/wheezing. Nevertheless, the significance of these associations is inconsistent among the included studies and any interpretation of the results should be drawn cautiously. Stratifying studies by pollutant reduced the number of eligible studies per pollutant and made a meta-analysis unfeasible.

Early childhood exposure to TRAP has significant impacts on lung development [16]. Starting from the early postnatal period till about 1.5 years of age, bulk alveolar formation in the lungs leads to substantial structural remodeling of the lung parenchyma [26, 29]. Microvascular maturation in the lungs also occurs starting from the early postnatal period till about 2-3 years of age. Damage to the lungs during the developmental periods has been associated with the development of long-term sequelae [27, 30, 31]. Additionally, compared to adults, children are more likely to be outdoors and active, have a higher ventilation rate, and are more likely to inhale pollutants into the distal lung, with accordingly higher exposure to TRAP [32].

NO_2_, PM_2.5_, and PM_10_ have been reported to be the primary constituents of TRAP [33], and long-term exposure to these pollutants in mice has been shown to lead to elevated levels of interleukin-6, a proinflammatory cytokine associated with inflammation and pulmonary diseases such as asthma [33–36]. TRAP exposure has also been associated with elevated expression of the Clca3 gene [33]. In animal models, expression of Clca3 has led to mucous cell metaplasia and airway hyperreactivity, leading to the development of episodic recurrent airway obstruction [37–40]. As the mucous cell metaplasia developed, it was observed that Muc5ac was the primary airway mucin expressed, which is also characteristic of human asthma [36, 37]. Consequently, it has been suggested that the association of TRAP with asthma onset may be due to the expression of Clca3 [33].

Genetic factors may also in part explain the heterogeneity in asthma and wheezing phenotype results presented in this review. Among children exposed to NO_2_, those with either a GSTP1 rs1138272 or rs1695 single nucleotide polymorphism (SNP) were found to be at an increased risk for asthma in a study combining multiple birth cohorts [41]. Additionally, high exposure to diesel exhaust particles (DEP) in children with the GST-P1 Val^105^ polymorphism was associated with a high risk of persistent wheezing [42]. Thus, differing genotypes among those exposed to TRAP may lead to differences in asthma or wheezing phenotypic outcomes.

Childhood asthma is a complex disease that involves many genetic and environmental factors, as well as interplay between these factors. Male children are at higher risk of childhood asthma than females, although this is reversed after puberty [43–46]. Male sex has also been shown to modify the association between prenatal PM_2.5_ exposure and childhood asthma onset [47]. It is uncertain whether similar interactions between sex and other forms of TRAP exist for childhood asthma onset. Exposure to other allergens such as mites is also associated with childhood asthma and can modify the risk of childhood asthma associated with TRAP [48]. Other environmental exposures such as prenatal smoke exposure, home dampness, and prenatal acetaminophen use can modify the association of genetic risk factors with childhood asthma onset [49–51].

Several limitations of this systematic review must be acknowledged. Firstly, the low number of eligible studies makes it difficult to draw any firm conclusions. Given that we assessed each pollutant separately, the number of studies per pollutant was reduced even further. Secondly, the heterogeneity in phenotype definitions and in study follow-up length may be a source of bias. The original phenotypic definitions from the Tucson Children's Respiratory Study measured outcomes at ages of 3 and 6 to define wheezing phenotype [5]. Given the differing follow-up length across the available studies, a child's phenotypic classification may differ between studies based on the definition used. A child with wheezing at ages of 3 and 4 but not at age of 6 would be classified as transient wheeze by Gehring et al. but would be persistent wheeze under Nordling et al., as follow-up ends at age of 4. It is therefore important to account for these differences in phenotype definition between studies. Finally, all but two studies used parental reporting of wheezing or asthma symptoms via questionnaire response to report asthma or wheezing in children. Although standard, this may lead to recall bias in the results. These limitations suggest that further studies studying TRAP exposure and the onset of childhood asthma and wheezing phenotypes are warranted.

## 5. Conclusion

Based on the results of this systematic review, there is evidence to suggest an association between TRAP exposure and transient as well as persistent childhood asthma/wheezing phenotypes. Conversely, TRAP may not be associated with late-onset asthma/wheezing phenotype. However, results remain inconsistent among different studies. The low number of studies per pollutant, as well as the heterogeneity in study methods such as follow-up length and in the phenotypic definitions of asthma and wheezing used, indicates the need for further studies on this topic.

## Figures and Tables

**Table 1:** Characteristics of included studies.

Study Reference and setting	Study design	Age group	Participants included	Exposure assessment	Traffic related pollutants	Traffic related pollutants measured	Asthma assessment	Transient asthma/wheezing definition	Persistent asthma/wheezing definition	Late-onset asthma/wheezing definition	Adjustment variables	CASP comments
(Brauer et al., 2006), Utrecht, Netherlands [19]	Birth cohort (PIAMA)	Birth – 4 years	4146	LUR model	PM_2.5_, NO_2_	PM_2.5_ mean: 16.9, range: [13.5,25.2] *µ*g/m^3^; NO_2_ mean: 25.4, range: [12.6,58.4] *µ*g/m^3^	Parental reporting of asthma/wheeze	Report of wheezing at age of 3 but not at age of 4	Report of wheezing at age of 3 as well as at age of 4	No report of wheezing at age of 3 but wheezing reported at age of 4^a^	Sex, study arm, allergic mother/father, mother/father's education, maternal smoking during pregnancy, breastfeeding at 3 months, gas stove, unvented gas water heater, siblings at birth, smoking at home, dampness in living room/child's bedroom, pets, daycare attendance, Dutch nationality, moving houses before age of 8	Pollutant levels only measured for four 2-week periods in a single year, risk of recall bias, no adjustment for familial history of asthma, race, or socioeconomic status (outside of education)

(Gehring et al., 2010), Utrecht, Netherlands [14]	Birth cohort (PIAMA)	Birth – 8 years	3863	LUR model	PM_2.5_, NO_2_	PM_2.5_ mean: 16.9, range: [13.5, 25.2] *µ*g/m^3^; NO_2_ mean: 25.4, range: [12.6,58.4] *µ*g/m^3^	Parental report of wheezing	Report of wheezing before age of 3 but no wheezing after age of 6	Report of wheezing before age of 3 as well as after age of 6	No report of wheezing before age of 3 but wheezing at age of 6 or later	Sex, study arm, allergic mother/father, mother/father's education, maternal smoking during pregnancy, breastfeeding at 3 months, gas stove, unvented gas water heater, siblings at birth, smoking at home, dampness in living room/child's bedroom, pets, daycare attendance, Dutch nationality, moving houses before age of 8	Pollutant levels only measured for four 2-week periods in a single year, risk of recall bias, no adjustment for familial history of asthma, race, or socioeconomic status (outside of education)

(Nordling et al., 2007), Stockholm, Sweden [20]	Birth cohort (BAMSE)	Birth – 4 years	3515	Dispersion model	PM_10_, NO_X_	PM_10_ mean: 3.9, 5^th^ – 95^th^ percentile: [0.94, 6.8] *µ*g/m^3^; NO_X_ mean: 23.1, 5^th^ – 95^th^ percentile: [4.7,48.7] *µ*g/m^3^	Parental report of wheezing	At least 3 episodes of wheezing before age of 2 but no episodes between ages of 3 and 4	At least 1 wheezing episode before age of 2 and at least 1 wheezing episode between ages of 3 and 4	No episode of wheezing before age of 2 but at least 1 episode of wheezing between ages of 3 and 4	Municipality, socioeconomic status, heredity, mother's smoking during pregnancy and infancy, year that house was built, damp or mold in the home at birth, and sex of the child	Risk of recall bias, no adjustment for race, endpoint is early for persistent asthma diagnosis

(Oftedal et al., 2009), Oslo, Norway [21]	Birth cohort (Oslo)	Birth – 10 years	2871	Dispersion model (EPISODE)	NO_2_	NO_2_ range: (1.4, 65.1), mean: 25.3 *µ*g/m^3^;	Parental reporting of doctor-diagnosed asthma/wheeze	None	None	Onset of doctor-diagnosed asthma after age of 4 years	Sex, parental atopy, maternal smoking in pregnancy, paternal education, and maternal marital status at the child's birth. Parental atopy was defined as a history of maternal or paternal asthma, hay fever, or eczema	Risk of recall bias, no adjustment for race nor socioeconomic status (except education and marital status)

(Pennington et al., 2018), Atlanta, Georgia, USA [22]	Birth cohort (KAPPA)	Birth – 6 years	24 608	Dispersion model (RLINE)	CO, PM_2.5_, NO_X_	CO median: 0.59 ppm; NO_x_ median: 55.5 ppb PM_2.5_ range: (0.06, 13.8), median 1.55 *µ*g/m^3^	At least one doctor diagnosis of asthma and one asthma-related medication dispensing after the first year of life from medical records	None	Evidence of incident asthma who also had evidence of asthma in the past year at each follow-up age up to age of 5 years	None	Sex, race, ethnicity, maternal asthma, maternal age, parental education, maternal marital status, neighborhood socioeconomic status (SES), birth year, and city region	Results presented as absolute risk difference, difficult to interpret

(Rancière et al., 2017), Paris, France [23]	Birth cohort (PARIS)	Birth – 4 years	3840	Dispersion model (Extra Index)	NO_x_	NO_2_ range: (39.0, 257.0), median: 75 *µ*g/m^3^	Parental reporting of doctor-diagnosed asthma or wheezing in the past 12 months at ages of 1, 2, 3, and 4	Wheezing occurring between 0 and 2 years of age and not till age of 4	Wheezing occurring between 0 and 2 years of age and persisting till age of 4	Wheezing occurring between 2 and 4 years of age	Sex, birth weight, family socioeconomic status, maternal education level, maternal history of asthma, allergic rhinitis, or eczema, paternal history of asthma, allergic rhinitis, or eczema, maternal smoking during pregnancy, exposure to environmental tobacco smoke at home during the first year, exclusive breastfeeding during the first 3 months, type of child care during the first 6 months, stressful family events during the first 2 years, body mass index ≥ 85th percentile for age and sex at 2–3 years, use of gas for cooking or heating in the home, and visible mold in the home	Potential for recall bias, no adjustment for race, endpoint is early for persistent wheezing diagnosis

(Sbihi et al., 2017), Vancouver, British Columbia, Canada [4]	Birth cohort	Birth – 10 years	68 195	LUR model	NO_2_, PM_2.5_	NO_2_ range: (15.0, 53.7), median: 33.3 *µ*g/m^3^; PM_2.5_ range: (3.2, 7.6), median: 5.4 *µ*g/m^3^	At least two primary care physician diagnoses within a 12-month period or a minimum of one hospital admission was identified as asthma cases each year	Asthma definition is met by age of 1 with asthma prevalence peaking among the group by age of 2 and no asthma activity after age of 6^b^	Asthma develops by age of 3 with asthma prevalence peaking among the group by age of 4 that is sustained until the end of follow-up^b^	Asthma develops by age of 3 with asthma prevalence peaking among the group by age of 6 and is sustained until the end of follow-up^b^	Sex, parity, breastfeeding initiation, birth weight, delivery mode, maternal smoking and educational attainment, and household income	Did not adjust for familial history of asthma, race, ethnicity Odds ratios reported only to 1 decimal place, only study to find an association between TRAP and late-onset asthma

^a^No result on the association between pollutant exposure and late-onset asthma phenotype was reported.

^b^Asthma phenotypes were defined based on group based trajectory modelling.

**Table 2:** Effect of TRAP and childhood asthma phenotypes in included studies.

Study reference and setting	Traffic related pollutant	Transient asthma/wheezing^a^	Persistent asthma/wheezing^a^	Late-onset asthma/wheezing^a^
Brauer et al., 2006 (Total n = 4146; Pollutant Exposure n = 2588) [19]	PM_2.5_	**OR: 1.16, 95**%** (CI: 1.00 - 1.34 per 4.4 **µ**g/m**^**3**^** increase of PM**_**2.5**_	OR: 1.19 (95% CI: 0.96 - 1.48) per 3.3 *µ*g/m^3^ increase of PM_2.5_	None
	NO_2_	**OR: 1.13 (95**%** CI: 1.00 - 1.28) per 10.6 **µ**g/m**^**3**^** increase of NO2**	OR: 1.13 (95% CI: 0.99 - 1.29) per 10.4 *µ*g/m^3^ increase of NO_2_	None

Gehring et al., 2010 (n = 3863; NO_2_ n = 2668) [14]	PM_2.5_	**OR: 1.29 (95**%** CI: 1.04 - 1.62) per 3.2 **µ**g/m**^**3**^** increase of PM**_**2.5**_	OR: 1.37, 95% CI: 0.99 - 1.91 per 3.2 *µ*g/m^3^ increase of PM_2.5_	**OR: 1.18, 95**%** CI: 1.01 - 1.37 per 3.2 **µ**g/m**^**3**^** increase of PM**_**2.5**_
	NO_2_	OR: 1.17 (95% CI: 0.97 - 1.41) per 10.4 *µ*g/m^3^ increase of NO2	OR: 1.30 95% (CI: 0.99 - 1.72) per 10.4 *µ*g/m^3^ increase of NO2	OR: 1.13 95% (CI: 0.99 - 1.35) per 10.4 *µ*g/m^3^ increase of NO2

Nordling et al., 2007 (n = 3515) [20]	PM_10_	OR: 0.90 (95% CI: 0.45 - 1.81) per 6 *µ*g/m^3^ increase of PM_2.5_	OR: 1.64 (95% CI: 0.90 - 3.00) per 3 *µ*g/m^3^ increase of NO_x_	OR: 0.94 (95% CI: 0.42 - 2.11) per 6 *µ*g/m^3^ increase of PM_2.5_
	NO_X_	OR: 0.82 (95% CI: 0.48 - 1.40) per 44 *µ*g/m^3^ increase of NO_x_	**OR: 1.60, 95**%** (CI: 1.09 - 2.36) per 44 **µ**g/m**^**3**^** increase of NO**_**x**_	OR: 0.87, 95% (CI: 0.47 - 1.60) per 44 *µ*g/m^3^ increase of NO_x_

Oftedal et al., 2009 (n= 2871, NO_2_ n = 2329) [21]	NO_2_	None	None	OR: 1.05 (95% CI: 0.64 - 1.72) per 27.3 *µ*g/m^3^ increase of NO2

Pennington et al., 2018 (Total n = 24 608; prenatal exposure n = 6795; 1^st^ year of life n = 7755) [22]	CO	None	**Prenatal exposure absolute risk increase: 3.5**%** (95**%** CI: 1.5**%**, 6.2**%**) per 2.7-fold increase CO**	None
			**Age 1 exposure absolute risk increase: 3.9**%** (95**%** CI: 1.5**%**, 6.2**%**) per 2.7-fold increase CO**	
	PM_2.5_	None	**Prenatal exposure absolute risk increase: 4.4**%** (95**%** CI: 2.3**%**, 6.4**%**) per 2.7-fold increase PM**_**2.5**_	None
			**Age 1 exposure absolute risk increase: 4.5**%** (95**%** CI: 2.3**%**, 6.6**%**) per 2.7-fold increase CO**	
	NO_x_	None	**Prenatal exposure absolute risk increase: 3.8**%** (95**%** CI: 1.7**%**, 5.9**%**) per 2.7-fold increase NO**_**x**_	None
			**Age 1 exposure absolute risk increase: 4.0**%** (95**%** CI: 1.8**%**, 6.1**%**) per 2.7-fold increase NO**_**x**_	

Rancière et al., 2017 (n = 3840, NO_x_ n = 698) [23]	NO_x_	OR: 1.03, 95% CI: 0.91 - 1.17 per 26 *µ*g/m^3^ increase of NO_2_ equivalent	**OR: 1.27, 95**%** CI: (1.09 - 1.47) per 26 **µ**g/m**^**3**^** increase of NO**_**2**_** equivalent**	OR: 1.19, (95% CI: 0.89 - 1.33) per 26 *µ*g/m^3^ increase of NO2 equivalent

Sbihi et al., 2017 (n = 68 195, NO_2_ n = 68 024) [4]^b^	NO_2_	**1 vs 0 OR: 1.10 (95**%** CI: 1.0 - 1.3)**	**1 vs 0 OR: 1.42 (95**%** CI: 1.1 - 1.8)**	**1 vs 0 OR: 1.18 (95**%** CI: 1.0 - 1.4) **
		2 vs 0 OR: 1.04 (95% CI: 0.9 - 1.2)	**2 vs 0 OR: 1.20 (95**%** CI: 1.0 - 1.5)**	**2 vs 0 OR: 1.29 (95**%** CI: 1.1 - 1.5). **
		**(3 vs 0 OR: 1.10 (95**%** CI: 1.0 - 1.3)**	3 vs 0 OR: 1.05 (95% CI: 0.9 - 1.2)	3 vs 0 OR: 1.00 (95% CI: 0.8 - 1.2).

	PM_2.5_	**1 vs 0 OR: 1.15 (95**%** CI: 1.0 - 1.3)**	**1 vs 0 OR: 1.17 (95**%** CI: 1.0 - 1.4) **	**1 vs 0 OR: 1.13, 95**%** CI: 1.0 - 1.3 **
		**2 vs 0 OR: 1.21 (95**%** CI: 1.1 - 1.4) **	2 vs 0 OR: 1.0 (95% CI: 0.8 - 1.2)	**2 vs 0 OR: 1.25, 95**%** CI: 1.1 - 1.5**
		3 vs 0 OR: 1.05 (95% CI: 0.9 - 1.2)	3 vs 0 OR: 0.86 (95% CI: 0.7 - 1.1)	3 vs 0 OR: 0.97, 95% CI: 0.8 - 1.1)

^a^Results in boldface indicate significant results at the 95% confidence level.

^b^Reported ORs compared higher exposure quartiles (groups 1, 2, and 3) to the lowest exposure quartile (reference group = 0). NO_2_ exposure ranged from 15 to 53.7 *µ*g/m^3^; PM_2.5_ exposure ranged from 3.2 to 7.6 *µ*g/m^3^.
